# Serum Oxytocin, Cortisol and Social Behavior in Calves: A Study in the Impossible Task Paradigm

**DOI:** 10.3390/ani13040646

**Published:** 2023-02-13

**Authors:** Claudia Pinelli, Anna Scandurra, Vincenzo Mastellone, Piera Iommelli, Nadia Musco, Maria Elena Pero, Alfredo Di Lucrezia, Daria Lotito, Raffaella Tudisco, Biagio D’Aniello, Federico Infascelli, Pietro Lombardi

**Affiliations:** 1 Department of Environmental, Biological and Pharmaceutical Sciences and Technologies, University of Campania “Luigi Vanvitelli”, 81100 Caserta, Italy; 2 Department of Biology, University of Naples Federico II, 80126 Naples, Italy; 3 Department of Veterinary Medicine and Animal Production, University of Naples Federico II, 80137 Naples, Italy; 4 Department of Pathology, Anatomy and Cell Biology, Columbia University, New York, NY 10032, USA

**Keywords:** behavior, cortisol, dairy cows, calves, human–animal interaction, impossible task, oxytocin

## Abstract

**Simple Summary:**

We investigated the relationship between circulating levels of the hormones oxytocin and cortisol and some behaviors directed at humans by calves in the impossible task paradigm. Cortisol correlates positively with the latency of behaviors directed at the caregiver and the duration of behaviors directed at the apparatus correlates negatively with people. Contrary to what is reported in the literature on cows, no correlations were found between oxytocin levels and direct behaviors toward the caregiver. This highlights a different behavioral strategy between calves and cows when faced with solving an impossible task.

**Abstract:**

In this study, we explored the correlations between circulating levels of oxytocin, cortisol, and different social behaviors toward humans in 26 Italian Red Pied calves (all females, with an average age of 174 ± 24 days) using the impossible task paradigm. This paradigm has proved fruitful in highlighting the effect of socialization on the willingness to interact with humans in several domesticated species. The test consists of the violation of an expectation (recovering food from an experimental apparatus) while a caregiver and a stranger are present. Immediately after the end of the test (less than one minute), blood was collected from the coccygeal vein. Statistics were performed by the Spearman’s rank correlation; significant differences were adjusted according to Bonferroni’s correction. Cortisol correlates positively (ρ = 0.565; *p* < 0.05) with the latency of behaviors directed at the caregiver, and the duration of behaviors directed at the apparatus correlates negatively with both the caregiver (ρ = −0.654; *p* < 0.05) and a stranger (ρ = −0.644; *p* < 0.05). Contrary to what is reported in the literature on cows, no correlations were found between oxytocin levels and direct behaviors toward the caregiver. This highlights a different behavioral strategy between calves and cows when placed in front of an impossible task.

## 1. Introduction

Scientific research on the human-animal bond is a rapidly expanding area of academic, clinical, and commercial interest. Concerning livestock animals, understanding domesticated animals’ psychological and social relationships, including with humans, can have significant implications for the quality of production [1,2] and animal welfare [3,4]. The domestication process of livestock was a milestone in the transition of society from nomadic hunters to agricultural communities, but it is possible that a close relationship between wild cattle and humans existed before [5]. Archaeological and genetic data are controversial, supporting two to three domestication events in cattle [6].

It is well known that hormones can act on behavior, which in turn arises from internal and environmental stimuli, with different stimuli eliciting different behaviors. Thus, circulating hormones often play a central role in regulating behavior and social relationships. Among the many hormones responsible for behavioral alterations in both humans and animals, a growing scientific interest occurred in hypothalamic neuropeptide oxytocin (OXT) functions, beyond its classical role in female reproduction [7,8,9,10]. In fact, for decades OXT has been suggested to act mainly, if not only, on the breast and uterus. During labor and delivery, it causes contractions of the uterine smooth muscle cells, favoring the expulsion of the fetus. During breastfeeding, the baby’s suction on the nipple stimulates the release of oxytocin, which in turn favors the contraction of the smooth muscles around the mammary glands, increasing the ejection of milk.

OXT is a highly conserved nonapeptide, produced primarily by the supraoptic and paraventricular hypothalamic nuclei and secreted in the neurohypophysis. It is present in all mammals with only minor changes [11]. Its involvement in the brain has been highlighted as a complex neuromodulator of a wide variety of behavioral and psychophysiological functions [12,13]. In non-human animals OXT affects sociability [14,15], determining and regulating intraspecific behaviors linked to sexual activity [16], couple-bonding [9,17], mother-offspring bonding [18,19,20,21], affiliative preferences [22,23,24], and alloparenting behaviors [25].

There is indication of a potential social-buffering effect of OXT: presence or contact with a mate subject reduces the effects of stressful events [26]. The physiological counterpart of social-buffering is the down-regulation of the activity of the hypothalamus-pituitary-adrenal (HPA) axis, and the decrease in cortisol levels, blood pressure, and heart rate [27,28]. All these physiological responses related to the release of OXT trigger a positive emotional state [29,30,31].

Other studies in both humans and other animals also support the hypothesis that OXT mediates interspecific relationships [10,32]. Basal levels of OXT in dogs are positively correlated with social gaze towards humans [33,34], and the dog’s gaze towards humans increases OXT in their owners [33]. Intranasal applications of OXT enhance the social bond between the owner and the dog [22], the dog’s ability to follow humans’ communicative signals [35,36], and the willingness of dogs to rescue their owners when they pretend to be trapped and stressed [37].

Among farm animals, ruminants interact commonly, and are able to establish a social bond, with humans [38]. Only a few studies addressed the intraspecific behavioral effects of circulating OXT in cattle [23,24] and there is only one interspecific study exploring the effect of OXT on social relation between humans and cows [39]. In the latter study, it was shown that circulating OXT was positively correlated with the duration and negatively correlated with the latency of animals’ interactions with the caregiver, providing support for the hypothesis of a relationship between OXT and the disposition of cows to engage in social contact with the caregiver. However, data in calves are missing, prompting this study. To this end, we used the impossible task paradigm (IT), which has proved fruitful in revealing the effect of socialization on the willingness to interact with humans in several domesticated species, such as dogs [40], goats [41], horses [42], and cows [39]. Therefore, in the current research, we replicated the study of D’Aniello et al. [39] to explore whether OXT is already effective in conditioning social contact with humans in calves. Accordingly, we measured the same behavioral parameters in the IT and performed the same hormone assays for plasma OXT. Considering the relationship between OXT and cortisol [43], we also dosed cortisol.

Serum OXT in cows positively correlates with social behavior with familiar subjects, both humans [39] and conspecifics [23,24]. However, such a relationship was not found between calves [23,24], indicating that OXT may be ineffective in regulating social relationships in early life. In the present study, we explored a possible correlation between serum OXT in calves and calves’ willingness to interact with humans.

## 2. Materials and Methods

### 2.1. Animals

Twenty-six Italian Red Pied calves (all females, with an average age of 174 ± 24 days), raised in a farm located in a hilly area of South Italy (Roccabascerana, Avellino, Italy) were included in the study.

According to farm practice, calves are born on the farm separated from the dam within 24 h of birth, then housed in a single stable (1.25 m × 1.48 m). The calves were spatially positioned next to each other and could have tactile and visual contact with neighboring calves. Each calf received mixed hay (composed by *Vicia sativa*, *Hedysarum coronarium*, *Avena sativa*, *Lolium multiflorum*, *Trifolium alexandrinum*) and alfalfa hay *ad libitum*. Additionally, the calves received 1.5 kg of calf starter meal composed of corn grain (57%) soybean (20%) and barley grain (16%) twice a day and a vitamin/mineral supplement (7%) produced by UVL Srl Roè Volciano (BS), Italy. Water was provided ad libitum by using water bowls.

The daily care of the animals (guidance and assistance during entry and exit from the shelter, nutrition, cleaning, and medical care) has been ensured by a caregiver. Ethical clearance was provided by the Ethical Animal Care and Use Committee of the University of Naples Federico II based on Italian Legislative Decree 26/2014 (art. 2).

### 2.2. Testing Procedure

The testing procedure was identical to that used in a recent study performed on cows from the same farm [39]. In brief, a specific experimental setup was built to induce a violation of the expectation in the calves. It consisted of a clear plastic container (30 cm circumference) whose inverted lid was fixed on a platform (60 cm × 40 cm) with screws. During the solvable tests, the container was put freely upside down on the lid; during the unsolvable trials, it was blocked. Three people participated in the test: the caregiver and two people unknown to the calves. One of them took the role of the stranger while the other one managed the apparatus and the feed during the test, moving away from the experimental area during the impossible trial.

The behavior of each calf was recorded onto a videotape through a SonyHDR-PJ260VE (Tokyo, Japan) camera placed in front of the calf’s head, at about 3 m. Other calves or people were banned from the testing area to avoid interference and influence on the behavior of the tested animals.

**Figure 1:**
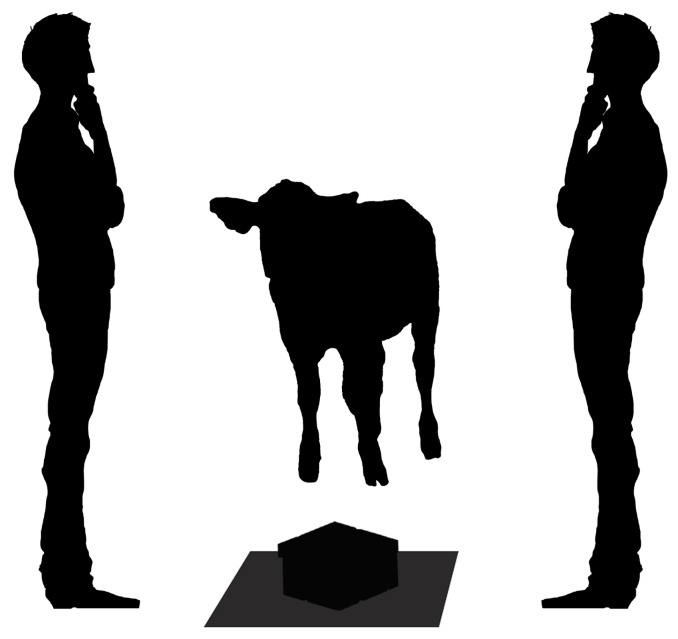
Schematic illustration of the experimental setting.

During the test, the apparatus was placed in front of the calves, while the caregiver and the experimenter were positioned on the lateral sides of the apparatus (Figure 1). The right-left position of the people was randomized.

In the training phase, which consisted of three consecutive solvable tests, the experimenter placed a palatable feed (corn flour) on the upside-down lid on the platform and covered it with the container without fixing it; the calf could reach the food easily by moving the container with its muzzle. During the impossible trial, the experimenter blocked the lid using the container, making the food unrecoverable. The test phase lasted 60 s. The caregiver and the stranger remained motionless, ignoring the calf throughout the unsolvable trial, even if solicited by the animal.

### 2.3. Behavioral Assessment

Behavioral analysis (Table 1) was performed through continuous sampling methods of video recordings using Solomon Coder^®^ software (Beta 16.06.26) (ELTE TTK, Budapest, Hungary) by a qualified researcher. The duration, frequency, and latency of each behavior was collected. The analysis included only the insolvable phase. The inter-observer reliability was determined by comparing the data to other obtained by another independent blind coder on 20% of the samples. The agreement between the two coders was extremely high for all behaviors (ranging from 96% to 99%).

### 2.4. Blood Sampling and Hormones Assay

Within one minute of the end of the test, 7 mL of blood was collected from the coccygeal vein in Vacutainer plastic tubes (Becton Dickinson, NJ, USA) and centrifuged at 4 °C and 1500 g for 15 min to obtain the serum. The serum was then aliquoted and frozen (−20 °C) until assayed. 

The serum concentrations of OXT and cortisol were measured by competitive enzyme immunoassay (EIA) according to the manufacturer’s instructions. An Oxytocin ELISA kit (Enzo Life Sciences, Lausen, Switzerland), validated for bovines [44], was used at a wavelength of 405 nm with a sensitivity ranging from 15.6 to 1000 pg/mL, intra-assay Precision: CV% < 8%, inter-assay precision: CV% < 10%. For the Bovine Cortisol ELISA Kit (MyBioSource, Inc., San Diego, CA, USA), the wavelength was set at 450 nm, the detection range was from 0.049 up to 200 ng/mL, and both intra- and inter-assay precision: CV% < 10% [45,46].

### 2.5. Statistical Approach

The Kolmogorov–Smirnov test reported most of the parameters not normally distributed. We therefore used a non-parametric statistical approach. The variables–OXT levels, cortisol levels, apparatus-directed behaviors (-DB), caregiver-DB, and stranger-DB–were used for analysis by Spearman’s rank correlation for the duration, the frequency, and the latency. Significant differences were adjusted according to Bonferroni’s correction. All statistical tests were performed with IBM SPSS statistical software 26 (IBM Corp., Armonk, NY, USA).

## 3. Results

Twenty-five calves out of 26 solved all the solvable trials and were therefore enrolled in the unsolvable phase. In total, 85% of animals interacted with the apparatus immediately after the start of the unsolvable trial.

**Figure 2:**
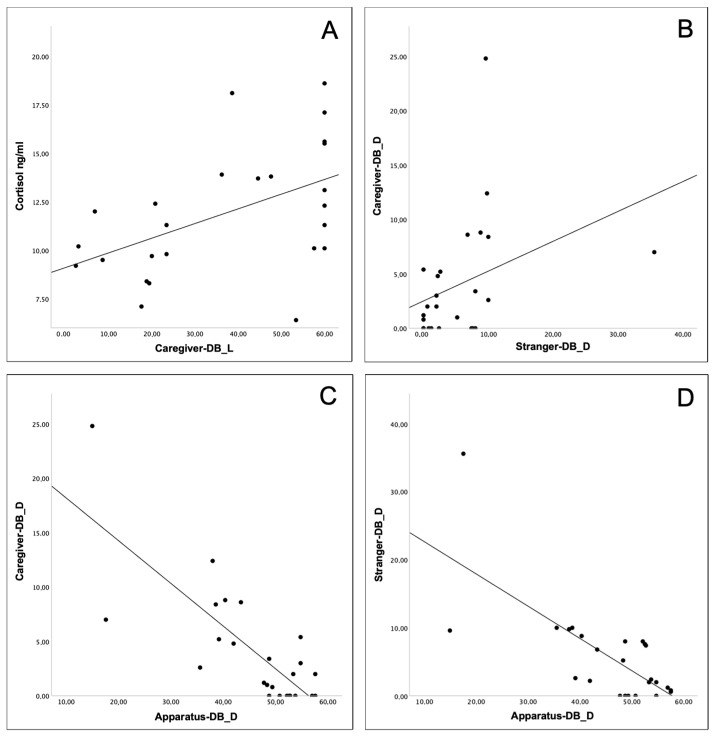
Spearman correlations: between cortisol and caregiver for latency (**A**); between caregiver and stranger for duration (**B**); between caregiver and apparatus (**C**), and stranger and apparatus (**D**) for duration. Sample size: 25 calves.

All the results of Spearman’s correlation analysis between OXT levels, cortisol levels, and behavioral variables (duration, frequency, and latency) of calves are presented in Table 2, Table 3 and Table 4. Significant results are shown in Figure 2.

While circulating OXT levels did not correlate with any of the caregiver- and stranger-DB (Table 2, Table 3 and Table 4), cortisol correlates positively with the latency of caregiver-DB (Table 4; Figure 2A).

A positive correlation between the duration of the caregiver- and the stranger-DB was observed (Table 2; Figure 2B) as well as a negative correlation between the duration of the apparatus-DB and both the caregiver- and stranger-DB (Table 2; Figure 2C,D).

## 4. Discussion

Previous data showed that OXT levels in cows correlated positively with the duration, and negatively with the latency, of interactions with the caregiver [39]. Cows showed more frequent, longer lasting, and quicker contact with the caregiver but not with the stranger [39]. Thus, elevated OXT levels are not associated with a general tendency to engage in social contact with humans but specifically with a familiar person. In calves, OXT levels did not correlate with any displayed behavior directed at humans, familiar or not. It is likely that the social behavioral pattern recorded in cows [39], besides being driven by hormones, is not genetically preconfigured but is acquired through socialization over time. This data is in line with our working hypothesis and with intraspecific studies underlining a correlation between OXT and social behaviors in cows but not in calves [23,24]. Taken together, these studies underline that the association between OXT and social behaviors in cows, both at intraspecific [23,24] and interspecific levels [39], is a functional system activated in adults. This conclusion is somewhat in contrast with studies showing that OXT is already active in early life as a key neuropeptide in the formation of mother–offspring bonds and as a possible social buffering mediator of HPA function [47]. Thus, the findings of the current paper point to the need to investigate the ontogenesis in other vertebrates in which OXT has been clearly linked to social behaviors in adults [33,48,49,50].

Previous research suggests an OXT-cortisol dynamic relationship, but the specific nature of this relationship and its context-specificity have not yet been fully clarified [51]. Some studies suggest that OXT increases may regulate HPA axis activity and buffer stress-induced release of cortisol, motivating prosocial behaviors like seeking support [52,53,54]. Instead, according to other research, cortisol can raise OXT [55]. In this study, no significant correlation between OXT and cortisol levels was detected, as also found in cows [39].

In calves, cortisol levels correlate positively with the latency of the interest in the caregiver. This means that cortisol induces a delay in interaction with the caregiver, but this effect was not observed in cows [39]. Ruminants commonly interact with humans, and their capacity to bond to humans has been shown [38]; they can also feel the perception of having a positive relationship with humans [56]. This feeling may provide the social buffering effect [26,57,58,59], which has been linked to the OXT in some studies [60,61]. The calves are likely not yet familiar with the caregiver, and so they cannot benefit from the social buffering effect.

The data shows a positive correlation between the duration of the behaviors directed toward the people involved in the test (i.e., caregiver and stranger), which in turn both negatively correlated with the duration of the interaction with the apparatus. These correlations were not observed in the cows [39]. This would indicate that calves do not discriminate between the caregiver and the stranger and do not consider whether they are familiar or not with people when busy solving the task. Interestingly, cows showed a positive correlation between the frequencies (unpublished data) of interaction with the apparatus and the caregiver [39]. This latter result could indicate an association between the familiar figure and the task they are trying to solve. Overall, the data in calves differ from those in cows [39], highlighting distinct behavioral strategies in juveniles and adults when faced with a problem.

This study presents a limitation. We were prevented from sampling the blood before the test to avoid a stressful procedure that could have affected behavioral outcomes. Several different kinds of specific stimuli can induce OXT release into the circulation andthe brain in response to the activation of sensory nerves. OXT may be released in response to tactile skin contacts (touch or stroking), sexual behavior, and feeding, as seen in primates [62], rodents [63], and dogs [64,65]. OXT is also released in response to positive social interaction [66]. Even visual contact of dogs [67] or artificially-reared sheep [68] with humans is sufficient to cause OXT increase. There is also evidence suggesting that the perception of the situation, rather than the action itself, may trigger OXT release [69,70,71]. Thus, it is not possible to be sure about the causal effect of OXT. In other words, it remains to be determined whether higher serum OXT levels can improve the cows’ behavior to seek social contact with the caregiver or if higher social contact raises serum OXT levels. However, it should be noted that, in the cited studies, the increase in OXT levels after social interactions was related to “feel good” activities, in which there was active human-mediated interaction. In our study, both the caregiver and the stranger were requested to be passive and unresponsive to any animals’ solicitation. In this condition, it was proved that this had no inducing effect on the OXT release [22,33,72]. Furthermore, previous studies in cows failed to find an effect on OXT in calves and cows before and after the manipulation of social interaction [24]. It is therefore more likely that OXT has a causal effect on social behavior, though more studies are needed to address this issue.

## 5. Conclusions

This study provides evidence that calves do not consider people when facing trouble in solving a task. Moreover, no indication that serum OXT could advantage social behavior toward humans was found. This outcome is somewhat different from that described in cows in which serum OXT positively correlates with social behavior with familiar humans [39]. This outcome, together with intraspecific studies [23,24], would indicate that the effect of OXT in favoring social behavior is not effective in early life. Thus, understanding the relation between OXT and social behavior during development may have important consequences in terms of production as well as for animal welfare. Further studies in other species during development are needed to shed light on the endocrine mechanisms underlying human-animal interaction.

## Figures and Tables

**Table 1:** Behaviors analyzed in the impossible task test. DB = directed behaviors.

Behaviour	Description
Apparatus-DB	Behaviours directed at the apparatus in an attempt to open it and reach food (e.g., touch, push, licking, biting, nibbling).
Caregiver-DB	Behaviours directed at the caregiver to gain physical contact with him (e.g., touch, push, licking, biting).
Stranger-DB	Behaviours directed at the stranger to gain physical contact with him (e.g., touch, push, licking, biting).

**Table 2:** Spearman correlations between oxytocin, cortisol, and behavioral variables (duration). Asterisks indicate a significant correlation. Sample size: 25 calves.

	Oxytocin		Cortisol		Apparatus-DB		Caregiver-DB		Stranger-DB	
	coef.	*p*-value	coef.	*p*-value	coef.	*p*-value	coef.	*p*-value	coef.	*p*-value
Oxytocin	1									
Cortisol	0.219	1	1							
Apparatus-DB	−0.023	1	0.403	0.183	1					
Caregiver-DB	−0.268	0.781	−0.478	0.063	−0.654	0.002 *	1			
Stranger-DB	0.040	1	−0.172	1	−0.644	0.002 *	0.496	0.047 *	1	

*: *p* < 0.05.

**Table 3:** Spearman correlations between oxytocin, cortisol, and behavioral variables (frequency). Asterisks indicate a significant correlation. Sample size: 25 calves.

	Oxytocin		Cortisol		Apparatus-DB		Caregiver-DB		Stranger-DB	
	coef.	*p*-value	coef.	*p*-value	coef.	*p*-value	coef.	*p*-value	coef.	*p*-value
Oxytocin	1									
Cortisol	0.219	1.168	1							
Apparatus-DB	−0.089	1	−0.179	1	1					
Caregiver-DB	−0.147	1	−0.288	0.651	0.182	1				
Stranger-DB	−0.040	1	0.031	1	0.175	1	0.330	0.428	1	

**Table 4:** Spearman correlations between oxytocin, cortisol, and behavioral variables (latency). Asterisks indicate a significant correlation. Sample size: 25 calves.

	Oxytocin		Cortisol		Apparatus-DB		Caregiver-DB		Stranger-DB	
	coef.	*p*-value	coef.	*p*-value	coef.	*p*-value	coef.	*p*-value	coef.	*p*-value
Oxytocin	1									
Cortisol	0.219	1	1							
Apparatus-DB	0.067	1	0.280	0.704	1					
Caregiver-DB	0.248	0.928	0.565	0.013 *	−0.086	1				
Stranger-DB	0.071	1	−0.106	1	−0.044	1	0.256	0.868	1	

*: *p* < 0.05.

## Data Availability

The datasets generated during and/or analyzed during the current study are available from the corresponding author upon reasonable request.
